# Retraction Note: Knowledge graph driven medicine recommendation system using graph neural networks on longitudinal medical records

**DOI:** 10.1038/s41598-026-36981-6

**Published:** 2026-01-26

**Authors:** Rajat Mishra, S. Shridevi

**Affiliations:** 1 School of Computer Science and Engineering, Vellore Institute of Technology - Chennai, Chennai, India; 2 Centre for Advanced Data Science, Vellore Institute of Technology - Chennai, Chennai, India

Retraction of: *Scientific Reports* 10.1038/s41598-024-75784-5, published online 26 October 2024

Editors have retracted this Article.

Following publication, concerns were raised with the Editors about the substantial similarity of this article with an arXiv preprint from a different set of authors^1^, which predates the publication of this Article. The Editors therefore consider this work redundant.

All authors disagree with this retraction.
